# Editorial: Entrainment of Biological Rhythms

**DOI:** 10.3389/fphys.2021.757000

**Published:** 2021-09-13

**Authors:** Rodolfo Costa, Charalambos Kyriacou

**Affiliations:** ^1^Department of Biology, University of Padua, Padua, Italy; ^2^Neuroscience Institute of the Italian National Research Council (CNR), Padua, Italy; ^3^Department of Genetics and Genome Biology, University of Leicester, Leicester, United Kingdom

**Keywords:** circadian rhythms, entrainment, environmental “zeitgebers”, biological rhythms, biological clocks

Circadian rhythms are ubiquitous and are observed in nearly all organisms that have been studied which inhabit our planet. These include animals, plants, fungi as well as some bacteria. These endogenous clocks will tick along nicely with an approximately 24 h period in constant conditions because they are generated by the so-called “transcriptional-translation-feedback-loops” (TTFLs) that were first described in Drosophila. The pioneers of this fly research, Jeff Hall, Michael Rosbash, and Mike Young were awarded the 2017 Nobel Prize in Medicine or Physiology reflecting the importance of their contribution to understanding a fundamental feature of life, namely, rhythmicity. These TTFLs appear to be bolted on and interconnected to a more primitive metabolic oscillator, which, under certain conditions, can be observed in the absence of the TTFL in a number of model species (Edgar et al., 2012).

While these oscillators are endogenous, their major role is presumed to be in anticipating the regular fluctuations in the environment that are associated with the Earth spinning on its axis every 24 h and preparing the organism for its daily chores. For animals this might mean foraging, finding a mate, outwitting predators and avoiding extreme daily heat or cold. In addition there are other geophysical cycles to which organisms are sensitive, for example the seasonal changes caused by the Earth's tilt around its axis as it circles the Sun. There is also the gravitational pull of the Moon on the oceans which generates 12.4 h cycles in the ebb and flow of the tides to which shoreline algae, animals and plants have responded by evolving circatidal cycles of behaviour and physiology. Other lunar-related cycles include semi-lunar (~15 days) and lunar cycles (29.5 days) which have important implications for the reproductive cycles of many organisms. All of these cycles, circadian and non-circadian entrain to environmental “Zeitgebers” (time-givers) the most important of which is the light cycle, but temperature cycles, social stimuli, seasonal photoperiodic changes, vibration or water pressure changes can be equally effective in “entraining” a biological clock to its optimal phase. It is entrainment that determines the time of day or chronotype, yet this property of circadian clocks is less well understood than free running rhythm.

This special edition of *Frontiers* is dedicated to “entrainment” in all its various forms. There are two extensive Drosophila reviews, one by George and Stanewsky, who focus on temperature entrainment of the fly clock and how the peripheral sense organs send temperature information to the brain and the other by Mazzotta et al. who review the neurogenetic basis for the effects of light on sleep in the fly. Light and temperature entrainment is developed further by Kaniewska et al. in the linden bug *(Pyrrhocoris apterus)*, a relatively new model for seasonal biology in which environmental inputs to the circadian clock have yet to be explored.

In flies, the original M and E hierarchical oscillator neurogenetic model has dedicated M (morning) clock neurons that are important for the morning burst of locomotor activity at dawn and the E (evening) neurons determine the late afternoon activity before lights off. By expressing the canonical clock protein PER in only M or E neurons, Menegazzi et al. show that under changing photoperiods the flies had difficulty in regulating their M or E activity at the appropriate times. This adds to the growing body of evidence that the M and E clock neurons are required to act as interacting network to generate normal locomotor activity, particularly under the more natural photoperiodic conditions found in temperate regions. The clock network is further studied by Van De Maas de Azevedo et al. who show that glutamate signalling from dorsal neurons in the clock network is required to generate the normal response to constant light, which is arrhythmicity. This also has implications because under natural conditions Drosophila species in the extreme northern hemisphere are not (nor need to be) strongly rhythmic under very long daylengths (Bertolini et al., 2019).

This theme of extreme environmental conditions such as constant light or long or short photoperiods that are prevalent in polar regions is the focus of the contribution by Appenroth et al. who study the High Arctic Svalbard ptarmigan (*Lagopus muta hyperborea*) and by Carmona et al. who study the effects of photoperiodic chronodisruption on pregnant female rats and the lingering effects this has on gene expression in their male progeny. In the former study, by investigating core body temperature and locomotor activity cycles, the authors reveal that the ptarmigan shows very weak rhythms, if at all, in both phenotypes under both constant light (LL) and constant darkness (DD) but are rhythmic under both long and short photoperiods under light-dark (LD) cycles. As shown in studies of reindeer, polar animals need not stay rhythmic under arrhythmic environmental conditions (Lu et al., 2010). In Carmona's et al. paper, pregnant female rats exposed to repeated abrupt reversals of the LD cycle gave birth to males, who at 90 days of age showed not only altered liver clock gene expression compared to animals who had gestated in an unchanging LD cycle, but also in genes that are risk factors for cardiovascular disease. There are clear medical implications for chronodisruption during pregnancy.

In zebrafish, unlike mammals, it has been long established that peripheral tissues have light sensitive clocks (Whitmore et al., 2000). Zebrafish encode a bewildering array of opsin genes (>30), and Steindal and Whitmore reveal that one third of these are expressed in cell lines from early larvae and about half of these are clock-controlled. Canonical clock genes such as *Per1* can also be induced and their circadian cycle of expression phase shifted in these cells by a broad range of light wavelengths reflecting multi-opsin gene expression. Because the clock controls the cycles of opsin expression that feed light information into the clock, the clock in effect determines the input to the pacemaker, a concept termed the “Zeitnehmer” (“time-taker”) that has been described in Neurospora (Merrow et al., 2003). The Zeitnehmer idea is followed up in the paper by Philippou et al. who use a chemical approach to disturb nuclear oscillations in Arabidopsis. Various chemical interventions implicate the electron transport chain in chloroplasts as being important for clock function. In particular, salicylic acid (SA) appears to be a signalling molecule that is gated by light in the first 3 h of the day and which can drive nuclear oscillations, thereby the light-mediated SA input to the clock modifies the gate by which SA influences the oscillation, i.e., input is output and *vice versa*.

Mathematical modelling of the clock and its environmental inputs is also represented by three papers. Ananthasubramaniam and Meijer use a probabilistic Markov model to study the rest-wake cycle of mice under both light dark (LD) and constant dark conditions (DD). They observe how light and the phase of the clock predict the animal's behaviour in the next time interval. Tokuda et al. use relatively simple amplitude-phase models in an attempt to reproduce the findings of vertebrate entrainment, jet-lag and seasonality experiments. One interesting finding from the model that is intuitively appealing is that the effects of jet-lag can be reduced when the amplitude of the rhythm is small, so resetting the clock phase is easier. The free-running endogenous period of any organism is not exactly set to the natural light cycle of 24 h. Schmal et al. incorporate a clock algorithm with a geophysical model of naturally varying seasonal light intensities and durations at different latitudes. Given an endogenous period and amplitude, the model predicts systematic seasonal and latitudinal changes in the phase on entrainment and the geographical distribution of chronotypes.

Switching to humans, Formentin et al. report a small-scale study from a hospital in Padova (Italy) where patients were fitted with glasses to give them bright light treatment in the morning and with lenses that filtered out blue light in the evening. They then assessed their sleep quality and mood which were generally improved in the treatment vs. control group. Finally, Roenneberg et al. argue the case for and against using Daylight Savings Time (DST), a complex political and social “hot potato.” They suggest that any decision needs to be based on biology and include social time, biological time and sun time. They favour an approach that dispenses with DST but realigns countries with their sun clock/body clock time. They also advocate a flexible approach in the workplace whereby subjects adjust their working time to that which best suits their body clock.

We would therefore like to thank all the authors for putting together such an interesting and varied collection of articles as well as the referees who helped improve the manuscripts. It was a pleasure to read and edit them, and we both learned a lot during the process.

## Author Contributions

Both authors listed have made a substantial, direct and intellectual contribution to the work, and approved it for publication.

## Conflict of Interest

The authors declare that the research was conducted in the absence of any commercial or financial relationships that could be construed as a potential conflict of interest.

## Publisher's Note

All claims expressed in this article are solely those of the authors and do not necessarily represent those of their affiliated organizations, or those of the publisher, the editors and the reviewers. Any product that may be evaluated in this article, or claim that may be made by its manufacturer, is not guaranteed or endorsed by the publisher.
